# Effect of drilling process parameters on bearing strength of glass fiber/aluminum mesh reinforced epoxy composites

**DOI:** 10.1038/s41598-023-39097-3

**Published:** 2023-07-26

**Authors:** Amr Seif, A. Fathy, A. A. Megahed

**Affiliations:** 1 Mechanical Design and Production Engineering Department, Faculty of Engineering, Zagazig University, P. O. Box 44519, Zagazig, Al-Sharqia Egypt; 2 Mechanical Department, Higher Technological Institute, Tenth of Ramadan City, Egypt

**Keywords:** Materials science, Materials for energy and catalysis, Structural materials, Techniques and instrumentation

## Abstract

The current study attempted to evaluate the impact of drilling parameters and delamination on the bearing strength of both neat GFRP (NG) and hybrid GFRP/aluminum (Al)-wire mesh with two various configurations, first with Al-mesh in the outer surface of specimen (AG) and the other with Al-mesh in the core of specimen (GA). Drilling procedure is carried out using $\varnothing$ 6 mm carbide twist drill with three different tip angles (90°, 120° and 135°), as well as Three different speeds and feeds (1000, 2000, and 3000 rpm) and (20, 40, and 60 mm/min), respectively. Taguchi and ANOVA analyses are used to analyze the influence of processing parameters. The findings showed that AG specimen experienced the least delamination damage. The maximum bearing strength refers to NG specimen, which is 9.6% and 8.7% more than AG and GA specimens, respectively. Drill point angle has the major effect on bearing strength for both AG and GA specimens, while for NG feed rate is of the major effect. The developed regression model displayed a high level of fitness with an average prediction error of less than 3%.

## Introduction

Recent years have seen a huge growth in the use of composite materials, particularly in the aerospace and aviation industries. This application focused on the requirement for substitute materials for steel and aluminum alloys that can lighten the structure's weight^1^. In this manner, a hybrid composite has been produced, combining the benefits of metal and fiber reinforced composites to create a superior hybrid composite known as fiber metal laminates (FML). The most frequently used metal in this is type of composites is aluminum^2^. FML families can be divided into many groups based on what reinforcement fiber used such as ARALL, CARALL and GLARE are abbreviations for aramid, carbon fiber and glass fiber, respectively^3^.The key benefit of FMLs over metal alloys is a better resistance to crack growth during fatigue because the fiber and polymers around the metal laminate function as a force-compressing mechanism that prevents crack initiation in the metal^4^. Additional features include the ability to manufacture complicated shapes utilizing various composite production processes, possible weight reduction and maintenance cost savings due to FMLs composites' strong corrosion resistance^5,6^. When metal wire meshes are employed instead of a sheet, it becomes possible to construct more complicated structures using the same manufacturing processes as for fiber reinforced composites. The metal mesh's capacity to plastically flex may be desirable in the event of an impact since it can postpone the fracture initiation and act as an additional energy absorber^7^, as well as improving bonding and limiting debonding downsides because it enhances the interfacial interaction between the resin and metal mesh which makes it more difficult to destroy the bonding between composite layers^8^. The addition of Al wire mesh boosts tensile and flexural elongation rates by up to 54% and 117%, respectively, and enhancing energy absorption^9^. These hybrid composites combine the best qualities of metal and FRPs, providing superior mechanical performance to traditional laminates. So, they can be employed in a variety of practical and crucial applications, including the military, transportation, aerospace, submarines parts, and other barrier applications^9,10^. These structures are linked together using mechanical connections such as rivets or bolts along with other methods. To assemble the structures, these joints required making holes. The hole quality, geometrical tolerance and material thickness have a significant impact on the joint's strength However, the most important factor is hole creation process or hole quality which results in a significant residual stresses around the hole boundary and reduces the structural strength. Moreover, poor hole quality accounts for 60% of parts that are refused during manufacturing^11^. Drilling of FML composite is a tough operation since the drill penetrates nonuniform structures that contain hard and abrasive fiber as well as the matrix that is heat sensitive, making the drilling process exceedingly problematic. Furthermore, drilling expenses are high owing to frequent regrinding of the drill bit due to significant erosion^12^. Although numerous studies have been done on drilling composite materials with a variety of methods, including molded and punched holes and innovative non-traditional techniques including laser and abrasive water jet, drilling is still the most common and simplest method for producing holes in laminate composites^13^. Conventional drilling results in a variety of problems, including internal cracks and delamination between laminates as well as heat damage, tool wear, and hole dimensions errors. These flaws compromise the drilled hole quality, which reduces the bolt connection's ability to support loads. Various researches have been done to examine how mechanical fasteners affect the bearing strength of laminate composites. The failure initiation that frequently happens in structural joints as a result of residual stress, fatigue, and fiber degradation brought on by drilling operations serves as the inspiration for these studies^14^. Delamination, which is defined as the dissociation of laminate layers that arises when the force acting among laminates is more than the interlaminar strength of the material, causes inter-ply breakdown, is generally considered to be the main damage to composite drilling^15^. Delamination is intrinsic to the assembly parts or bolt connections because it reduces the material's strength to withstand excessive loading^16^. Peel-up and push-out delamination, which are drill-induced delamination, are both presented at the entrance and departure of the holes^17^. According to Khashaba et al.^18^, push-out delamination is more severe than peel-up delamination. Controlling some significant aspects, such as drill tool material and geometry, cutting speed, feed rate and backup mechanisms is the key to minimize delamination when drilling composite laminates. These parameters impact the quality of the drilled hole and the drilling process. According to research by^19^, the delamination is directly connected to feed rate. The results demonstrated by Sakthivel et al.^20^ stated that feed rate is the most significant variable in drilling of Glass fiber Reinforced Stainless Steel Mesh Polymer composites. These findings agreed with research by Jenarthanan et al.^21^. Low spindle speeds result in less damage, however using a high speed with a low feed rate can mitigate delamination^22^. The impact of choosing tool geometry and operating conditions on drilling-induced damages was proven by the author, he indicates that a smaller tip angle^23^ and a lower feed rate were associated with holes that had less delamination based on his research. Where^24,25^ clarifies, with a smaller point angle the thrust force is reduced which accounts for the lower damage. The carbide drill is considered to be the best alternative for drilling composite than HSS drill because it creates less delamination and wear^26^. Poor drilling results in reducing bearing strength, and vice versa. According to^19^, feed rate has a major impact on bearing as low feed rate and high-speed enhance bearing strength. Different types of failure modes are generated by mechanically fastened joints, including net-tension, shearing-out, cleavage and bearing^27^, these fracture mechanisms are quite complex and are influenced by several factors, including washer dimension and lateral clamping force. Taguchi statistical method is well-suited for engineering optimization problems that require measuring response quality characteristics that deviate from stated standards using the S/N ratio^28,29^.

According to authors’ knowledge, no researches have focused on how drilling process variables and induced delamination affect the bearing strength of FML composites that use Al wire mesh as reinforcement rather than sheets or stainless-steel wire mesh. In this work, we explored the drilling of various specimen structures, such as neat glass fiber (NG) and specimens combining Al wire mesh and glass fiber in two different patterns, first having an Al-mesh in the outer layer (AG) and the second in the core (GA). Specimens were drilled with different drilling parameters (spindle speed, feed rate and drill tip angle). The current study's objective is to ascertain the impact of drilling conditions such as spindle speed (N), feed rate (F) and drill point angle (Ɵ) on delamination and bearing strength. Taguchi and ANOVA analyses were applied to assess and optimize operating parameters and their influence on bearing strength. Regression analysis was performed in relation to specified design variables to predict the ideal process parameter level and improving bearing strength.

## Materials and experimentation

### Specimen fabrication

**Figure 1:**
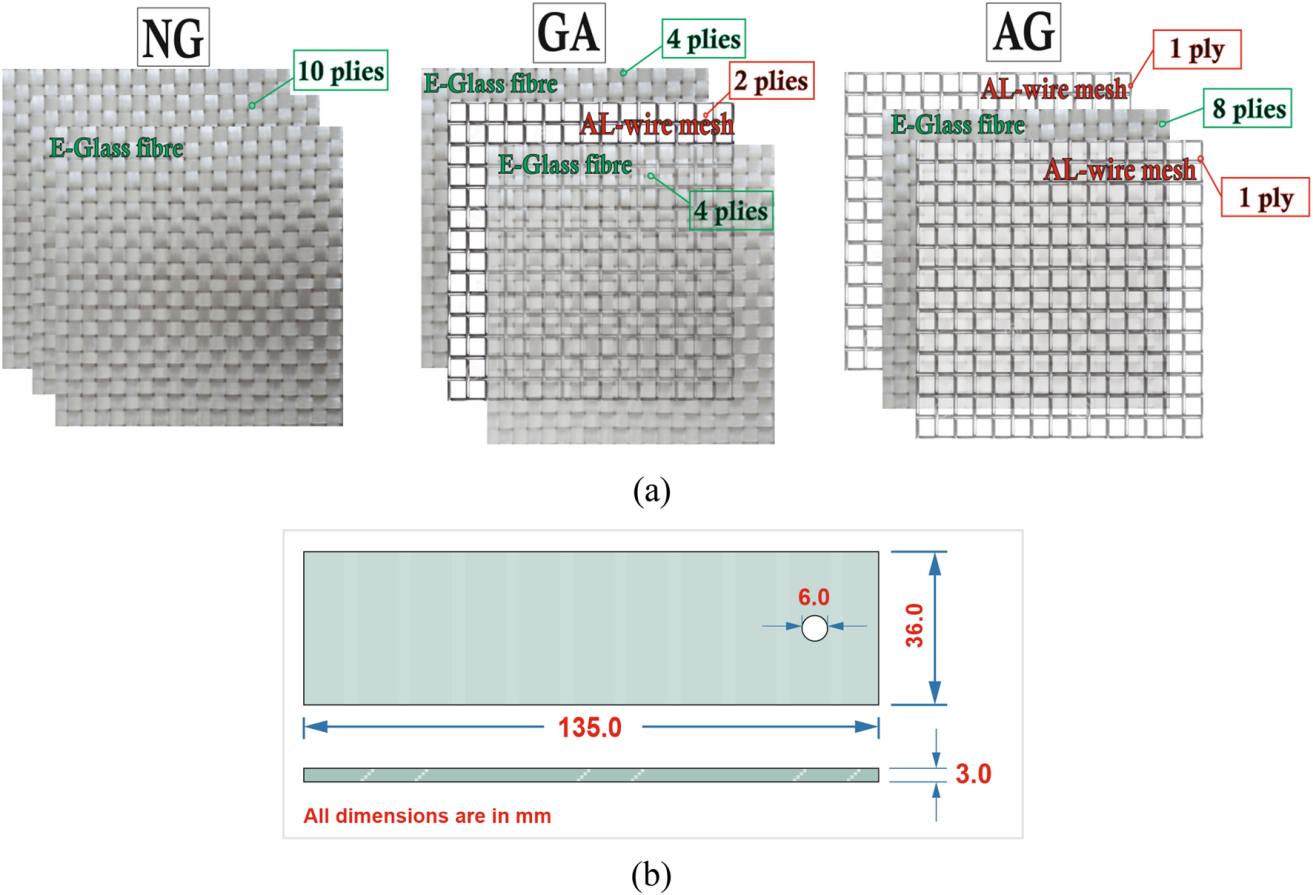
Manufactured specimens; (**a**) laminates’ sequence of NG, AG, and GA specimens, and (**b**) geometries of the test specimen.

In this study, three specimens with 3 $\pm$ 0.25 mm thickness were prepared from ten layers by utilizing the hand layup technique. First specimen is neat glass (NG) made of woven roving E-glass fiber and the other two hybrid composite laminates consist of E-glass fiber and aluminum wire mesh with alternative patterns. AG-specimen in which the Al-wire mesh placed in the outer layers and GA-specimen with Al-wire mesh placed in the core plies of specimen’s sequence as shown in Fig. 1a. The epoxy matrix consists of part A Biresin^®^ CR82 (resin) and part B CH80-6 (hardener) supplied by Sika Industry^30^. Details about the properties of glass fiber and epoxy are listed in Table 1. Specimens were then cut into coupons with dimensions of 135 × 36 × 3.0 ${\text{mm}}^{3}$ using CNC milling machine according to ASTM D5961^31^, Fig. 1b.

**Table 1:** Mechanical properties of E-glass fiber and epoxy as given by the supplier.

property	E-glass fiber	Epoxy
Density (g/cm^3^)	2.54	1.14
Young’s modulus (GPa)	76	2.9
Poisson’s ratio	0.22	–
Shear modulus (GPa)	33	–
Elongation (%)	1.8–3.2	6.4
Tensile strength (MPa)	3400	84
Fiber diameter (μm)	17 ± 2	–
Compressive strength MPa	–	110
Shore hardness –	–	D 85

### Drilling process

**Figure 2:**
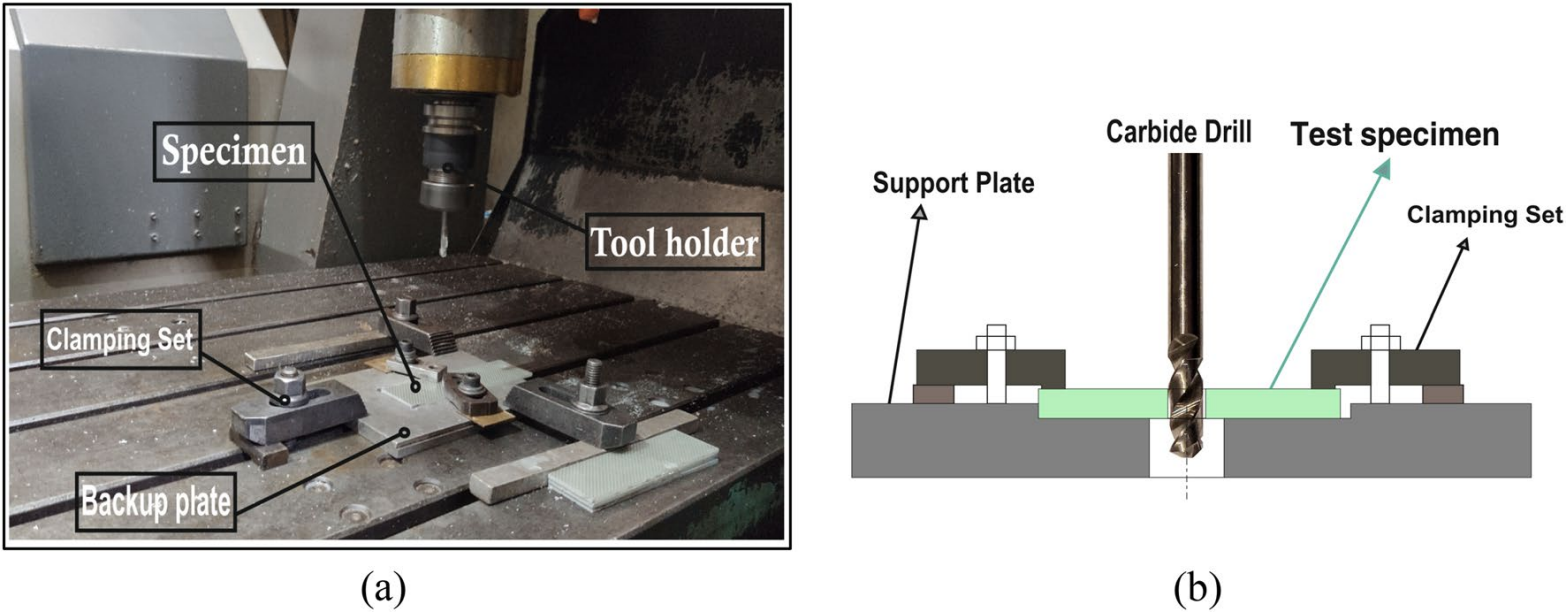
The experimental setup of; (**a**) the CNC drilling process*,* and (**b**) the backup plate.

The drilling process on the three specimens were implemented by using a BMDX8060 CNC milling machine which has spindle speeds up to 18,000 rpm. Specimens were drilled under dry cutting conditions; with three spindle speeds (1000, 2000 and 3000 rpm) and three feeds (20, 40 and 60 mm/min) using three solid carbide twist drills of 6 mm diameter with point angles of (90°, 120° and 135°). Table 2 illustrates information of drill materials and geometry. An aluminum backup plate was utilized to enhance the drilling process quality^32^. Setup of the drilling process is shown in Fig. 2.

**Table 2:** Geometries of the carbide drills.

D (mm)	6	
Number of fluted	2	
Flute length (mm)	24	
Overall length (mm)	67	
Helix angle	30°	
Clearance angle	10°	
Point angles	90°/120°/135°

### Experimental design

The experimental work was performed according to Taguchi L9 orthogonal array (OA)^28^, in which 3 factors with 3 levels were selected. Taguchi technique aids in investigation of the impact of all process parameters on responses by minimizing the number of tests and hence the cost of experiments. Taguchi technique requires less experiment or data to determine the best machining condition. Therefore, if the experimental run is time-consuming and expensive, it is advised to employ the Taguchi method^33^. Drilling parameters such as spindle speed, feed rate and drill point angle were selected in accordance with earlier literatures^18,20,24,25^ , the constraints of the CNC machine, and early test runs where some of the drilling process' variables are determined by experimentation. Table 3 shows the experiments’ factors and their levels. Also, the resulting L9-OA experimental design is summarized in Table 4.

**Table 3:** Independent factors with their respective levels.

Input factors	Symbol	Units	Level 1	Level 2	Level 3
Speed	N	rpm	1000	2000	3000
Feed rate	F	mm/min	20	40	60
Drill point angle	Ɵ	Degree	90	120	135

**Table 4:** L9 orthogonal array experimental parametric design.

Experiment no.	Speed (rpm)	Feed rate (mm/min)	Drill point angle (°)
1	1000	20	90°
2	1000	40	120°
3	1000	60	135°
4	2000	20	120°
5	2000	40	135°
6	2000	60	90°
7	3000	20	135°
8	3000	40	90°
9	3000	60	120°

## Assessment of the responses

### Assessment of delamination size

**Figure 3:**
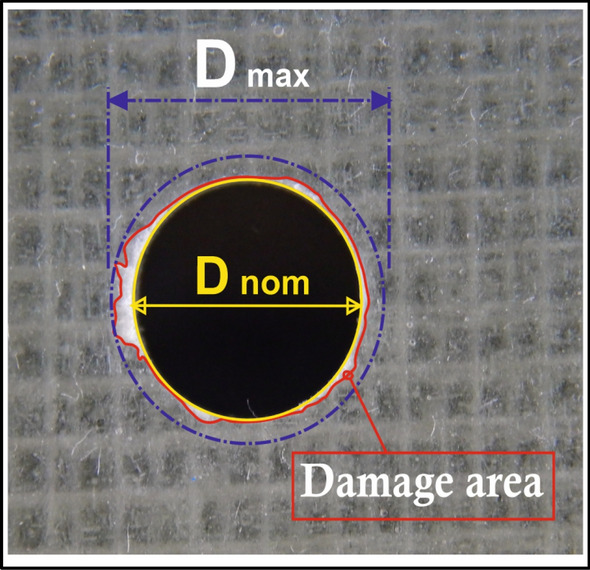
Digital image of drilled specimen.

Delamination (peel-up at hole entry and push-out at hole exit) were measured using a high-resolution camera with large optical zoom and then image processing using CorelDraw software to assess the maximum diameter of the delamination zone within resolution of ${10}^{-3}$ mm. Drilling damage was related to the equation of delamination factor (*F*_*d*_) presented by Chen^34^ as given in Eq. (1).

1 $${F}_{d}=\frac{{D}_{MAX}}{{D}_{NOM}}$$

where ${D}_{MAX}$ is the maximum diameter of the delamination area and ${D}_{NOM}$ is the nominal diameter of the hole (6 mm), as illustrated in Fig. 3.

### Assessment of bearing strength

**Figure 4:**
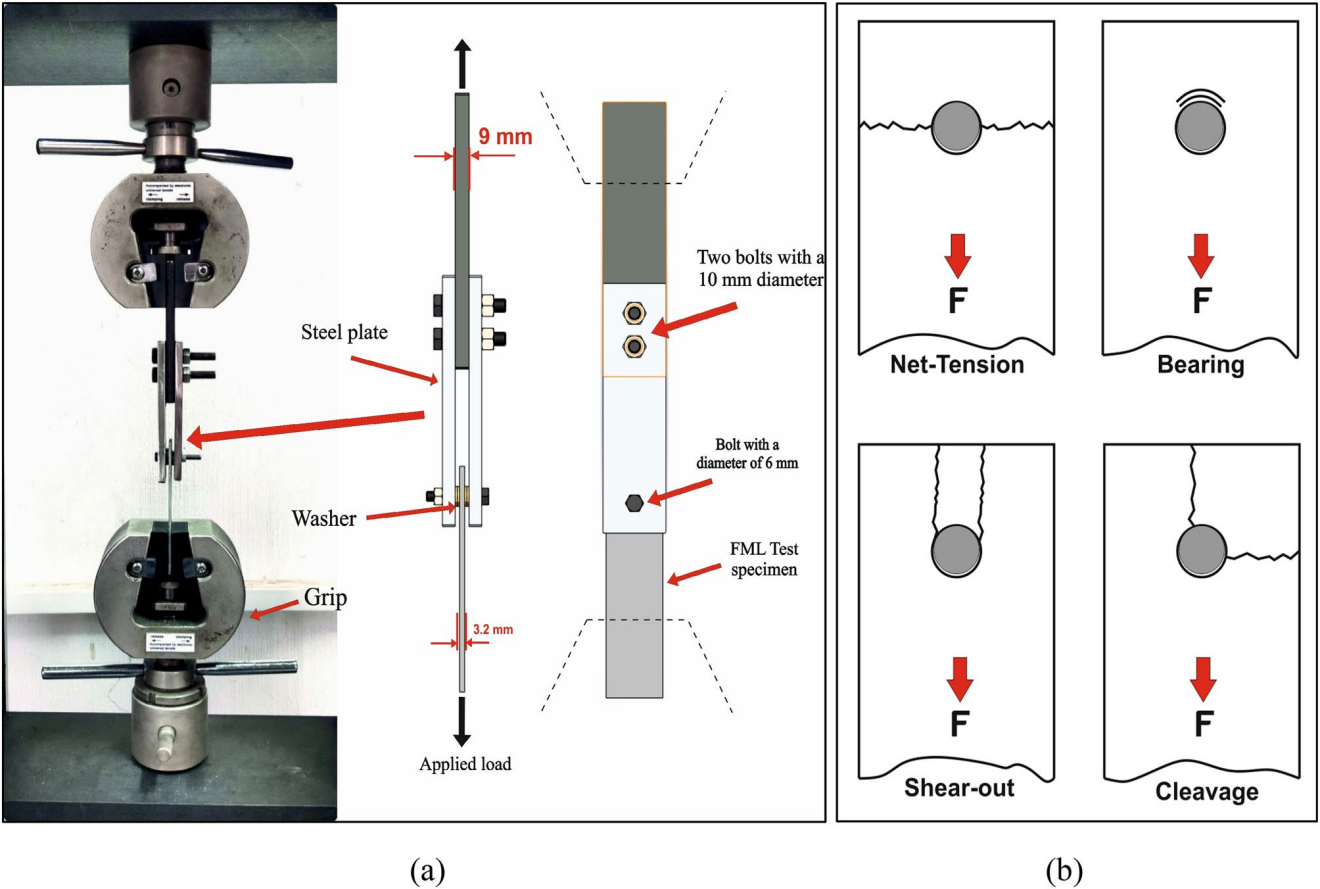
Bearing test; (**a**) experimental setup of the bearing strength test, and (**b**) failure modes of a mechanically fastened joint composite.

Bearing tests were performed according to ASTM D5961M standard^31^. The bearing specimens were cut out from the produced laminates sheet parallel to the fiber and wire orientation. Then drilling operations were performed on the specimens to make 6 mm hole. The test specimen was loaded at the hole by using steel pin and then normal application of bearing force through a lightly torqued fastener that undergo in double shear by a fixture like that shown in Fig. 4a. Clamping torque of 10 N.m was applied in tightening the bolted connection^35^. Loading of the assembly in tension in the test machine produces the bearing force.

The bearing test was carried out at room temperature in a computerized universal testing machine of a model (Jinan Test Machine WDW 100 kN) with 2 mm/min head speed till the load was dropped, the test machine setup is shown in Fig. 4a. The bearing strength value was obtained as the average of five tested specimens.

In mechanically fastened joint composite, the failure modes can be classified into four types^27^ net-tension, shear-out, bearing, or Cleavage failure that are indicated in Fig. 4b.

The determination of bearing stress was carried out by dividing the maximum bearing load by the bearing area^36^, according to Eq. (2). Where ${F}_{max}$ is the maximum load applied, ${D}_{h}$ is the hole diameter and t is the thickness of the sample.

2 $${\sigma }_{b}=\frac{{F}_{max}}{{D}_{h}\times t}$$

The bearing strain of the test specimen was calculated using Eq. (3)^31^.

3 $${\varepsilon }_{br }=\frac{{\delta }_{d}}{K\times D}$$

where ${\varepsilon }_{br}$ is the bearing strain, ${\delta }_{d}$ is the hole deformation (mm), *D* is the hole diameter (mm) and K is constant value 1 for double shear test, 2 for single shear test.

## Results and discussion

Table 5 summarizes the experimental observations for each setting used to estimate the influence of various machining parameters on the drilling response factors (delamination factor and bearing strength) and determine the optimal processing conditions to achieve the maximum quality for each response.

**Table 5:** The experimental results.

Specimen type	No	Machining parameters			Responses		
		Speed (rpm)	Feed rate (mm/min)	Drill point angle (°)	Push-out delamination (${F}_{d}$)	Peel-up delamination (${F}_{d}$)	Bearing strength (MPa)${ \sigma }_{b}$
Neat glass	1	1000	20	90°	1.10419	1.09042	328.69
	2	1000	40	120°	1.10844	1.09281	336.14
	3	1000	60	135°	1.14212	1.12950	323.89
	4	2000	20	120°	1.12659	1.09541	317.07
	5	2000	40	135°	1.14557	1.09820	346.19
	6	2000	60	90°	1.12182	1.08456	334.46
	7	3000	20	135°	1.12918	1.07220	323.39
	8	3000	40	90°	1.11537	1.08907	346.23
	9	3000	60	120°	1.11985	1.09503	341.67
AG	1	1000	20	90°	1.04081	1.03877	316.15
	2	1000	40	120°	1.05477	1.06192	288.4
	3	1000	60	135°	1.06361	1.06830	274.15
	4	2000	20	120°	1.06040	1.06088	298.7
	5	2000	40	135°	1.06142	1.05743	303.7
	6	2000	60	90°	1.05540	1.06113	328.4
	7	3000	20	135°	1.05661	1.06176	305.36
	8	3000	40	90°	1.05429	1.05084	311.9
	9	3000	60	120°	1.06108	1.06300	284.9
GA	1	1000	20	90°	1.11142	1.10332	314.68
	2	1000	40	120°	1.09608	1.13413	297.98
	3	1000	60	135°	1.14243	1.14388	284.22
	4	2000	20	120°	1.11760	1.08016	311.49
	5	2000	40	135°	1.12319	1.11537	300.18
	6	2000	60	90°	1.10314	1.11438	312.32
	7	3000	20	135°	1.12791	1.08967	279.13
	8	3000	40	90°	1.13000	1.10967	321.15
	9	3000	60	120°	1.14291	1.11218	316.83

### Effect of process parameters on delamination

In the present study, the signal-to-noise (S/N) ratio was calculated using the "smaller is better" technique^37^ represented in Eq. (4) to optimize the chosen process parameter with the goal of minimizing the delamination damage.

4 $$\text{Smaller\, is \, better}, \, \frac{S}{N}\text{ ratio }= -10 \, \text{ log }[ \frac{1}{n} \sum {y}_{i}^{2} ]$$

where *n* is the total number of observations, ${y}_{i}$ is the response value, and *i* varies from 0 to *n*.

#### (A) Peel-up delamination

**Figure 5:**
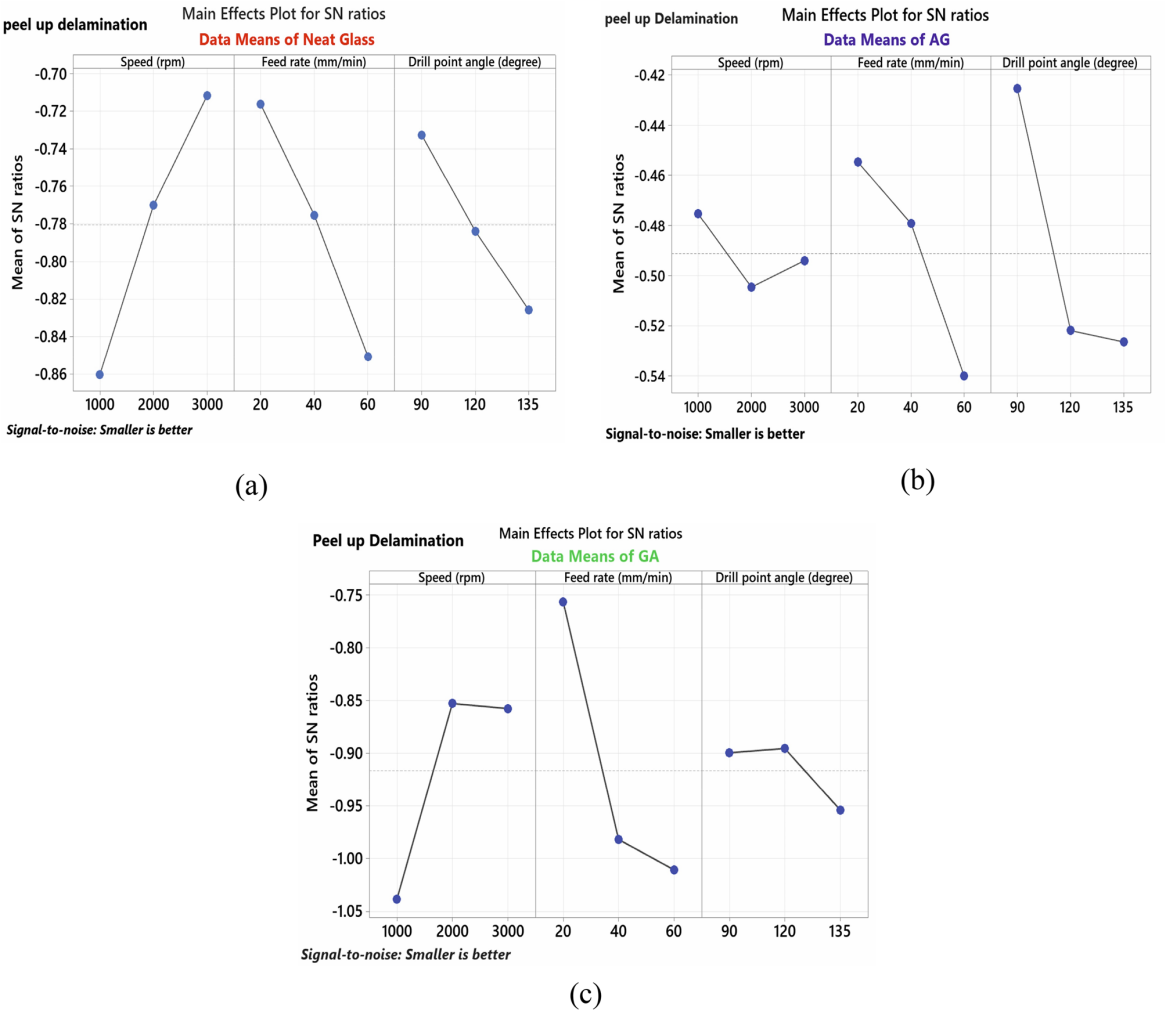
Main effect plot for S/N ratios of peel-up delamination for; (**a**) NG-specimen, (**b**) AG-specimen, and (**c**) GA-specimen.

From Taguchi analysis listed in Table 6 and indicated in Fig. 5, the optimum conditions which result in minimum peel-up delamination for NG specimen are spindle speed of 3000 rpm, feed rate of 20 mm/min and point angle of 90°, while speed has the major effect in peel-up delamination. Moreover, peel-up delamination rises with increasing feed and point angle while decreasing with increasing spindle speed as seen in Fig. 5a. For AG-specimen, speed of 1000 rpm with feed rate 20 mm/min and point angle 90° are the optimal conditions. Figure 5b also shows that the drill angle, followed by feed rate, has a significant impact on peel-up delamination, while spindle speed has a minor effect. Peel-up delamination rises with increasing feed rate, point angle and spindle speed up to 2000 rpm then starts to decrease. For GA-specimen, 2000 rpm speed with feed of 20 mm/min and point angle 120° resulted in the lowest damage. Feed rate has a major effect on peel-up delamination, followed by speed and point angle, which has a minimal impact. Peel-up delamination rises with increasing feed rate and point angle and reduces with increasing spindle speed as seen in Fig. 5c. In general, increasing of feed rate and drill point angle will increase peel-up delamination as a result of increasing the thrust force at the drill bit^19^. AG specimen shows the smallest peel-up delamination compared to the other two specimens.

**Table 6:** Response table for S/N ratio (minor is better) for peel-up delamination.

Specimen	Level	Speed (N)	Feed rate (F)	Drill point angle (Ɵ)
NG-specimen	1	−0.8602	−**0.7163**	−**0.7327**
	2	−0.7701	−0.7752	−0.7837
	3	−**0.7117**	−0.8504	−0.8256
	Delta	0.1484	0.1341	0.0930
	Rank	**1**	**2**	**3**
AG-specimen	1	−**0.4754**	−**0.4548**	−**0.4255**
	2	−0.5046	−0.4792	−0.5219
	3	−0.4940	−0.5400	−0.5265
	Delta	0.0292	0.0852	0.1010
	Rank	**3**	**2**	**1**
GA-specimen	1	−1.0383	−**0.7566**	−0.8995
	2	−**0.8529**	−0.9818	−**0.8955**
	3	−0.8577	−1.0106	−0.9540
	Delta	0.1854	0.2540	0.0585
	Rank	**2**	**1**	**3**

Bold values show the optimal levels of control variables.

#### (B) Push-out delamination

**Figure 6:**
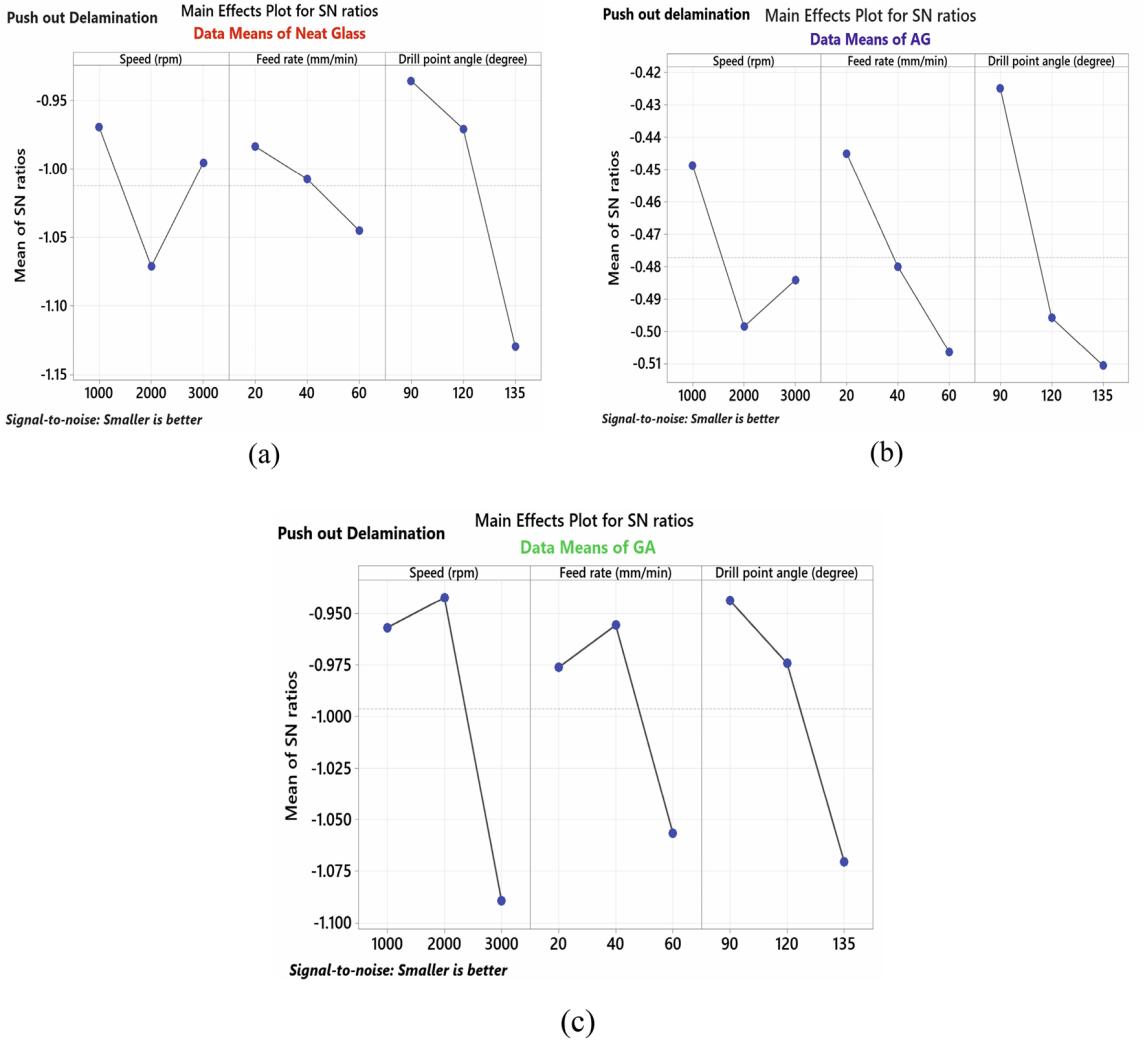
Main effect plot for S/N ratios of push-out delamination for; (**a**) NG-specimen, (**b**) AG-specimen, (**c**) GA-specimen.

**Figure 7:**
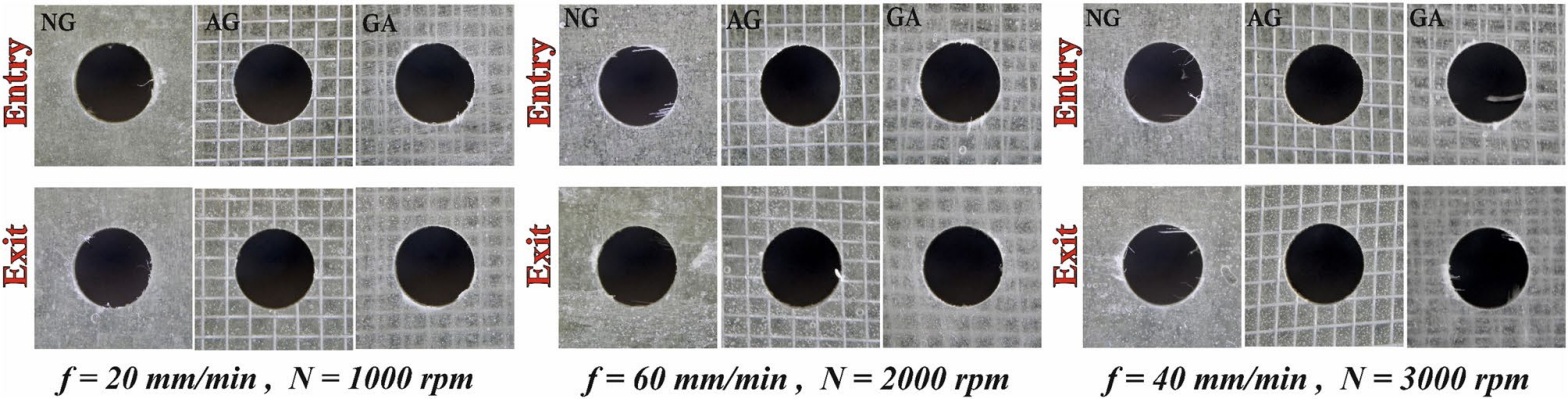
Selected photographs illustrating the effect of feed rate and spindle speed on delamination for point angle of 90°.

From the results of S/N ratio listed in Table 7 and main effect plot for S/N ratios of push-out delamination shown in Fig. 6, the optimal process conditions for minimizing damage are spindle speed of 1000 rpm, feed rate of 20 mm/min and point angle of 90° for both NG and AG-specimens. For GA the best combination is 2000 rpm spindle speed with 40 mm/min feed and 90°-point angle. The drill point angle is of major impact for both NG and AG specimens. While for GA specimen the parameter with the highest effect is the spindle speed. Figure 6a illustrates that raising the feed rate, point angle, and spindle speed up to 2000 rpm increases push-out delamination in NG-specimen owing to increasing thrust force existed during the drilling operation. When spindle speeds exceed 2000 rpm, push-out delamination begins to reduce. This may be related to the ease of removing material at high speeds and providing a smooth cutting surface, which is the same result attained by Khashaba and El-Keran^19^. Figure 6b shows that the lowest push-out delamination values in AG-specimen are obtained with lower spindle speeds, feed rates, and point angles. This is associated with a reduction in drilling push force. For GA-specimen, high spindle speed (3000 rpm), feed rate (60 mm/min), and point angle (135°) are related to the highest values of push-out delamination as shown in Fig. 6c since these conditions increase the drill tool's thrust force, which in turn increases delamination damages. The smallest push-out delamination is presented in AG, followed by GA and finally NG-specimen. Push-out delamination is more significant than peel-up delamination since it typically increases by approximately an average percent of 6%. Compared to NG and GA specimens, AG-specimen exhibits a relative improvement in both peel-up and push-out delaminations. This may be explained by the significantly better bonding between the Al wire mesh layer and the other layers below it in AG specimen relative to the slightly weaker bonding between the two adjacent Al wire mesh layers in the GA specimen. Figure 7 shows selected photographs of delamination at entry and exit sides of some drilled holes which illustrate the effect of different machining conditions.

**Table 7:** Response table for S/N ratio (minor is better) for push-out delamination.

Specimen	Level	Speed (N)	Feed rate (F)	Drill point angle (Ɵ)
NG-specimen	1	−**0.9698**	−**0.9838**	−**0.9359**
	2	−1.0714	−1.0077	−0.9709
	3	−0.9956	−1.0453	−1.1300
	Delta	0.1016	0.0615	0.1941
	Rank	**2**	**3**	**1**
AG-specimen	1	−**0.4487**	−**0.4450**	−**0.4250**
	2	−0.4985	−0.4800	−0.4958
	3	−0.4841	−0.5063	−0.5106
	Delta	0.0497	0.0613	0.0856
	Rank	**3**	**2**	**1**
GA-specimen	1	−0.9570	−0.9763	−**0.9439**
	2	−**0.9425**	−**0.9558**	−0.9743
	3	−1.0891	−1.0565	−1.0704
	Delta	0.1466	0.1007	0.1265
	Rank	**1**	**3**	**2**

Bold values show the optimal levels of control variables.

### Effect of process parameters on bearing strength

Bearing strength which is measured for each experiment combination is the average of five test specimens for each setup. The experimental data are observed and analyzed using Taguchi analysis to indicate the main effect of parameters on bearing strength and (S/N) ratio response for detecting the optimum process parameters. Analysis of Variance (ANOVA) is used to indicate the influence of machining parameters affecting the output responses and to identify which parameter(s) is(are) significant^20^. Analyses were implemented using Minitab 20 software. Taguchi analysis was chosen^37^ to survey the experimental data, the equation of ‘larger is better’ characteristic is presented in Eq. (5).

5 $$\text{Larger \, is \, better},\, \frac{S}{N}\text{ratio }= -10\, \text{ log}[ \frac{1}{n} \sum \frac{1}{{y}_{i}^{2}} ]$$

where n is the total number of observations, ${y}_{i}$ is the response value and i varies from 0 to n.

#### (A) NG specimen

**Figure 8:**
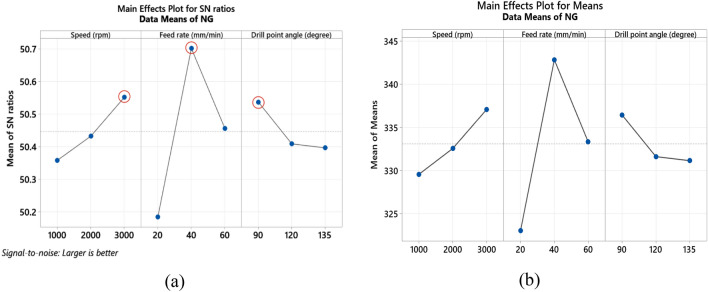
Main effects plot of NG specimen; (**a**) S/N ratios, and (**b**) means for bearing strength.

**Figure 9:**
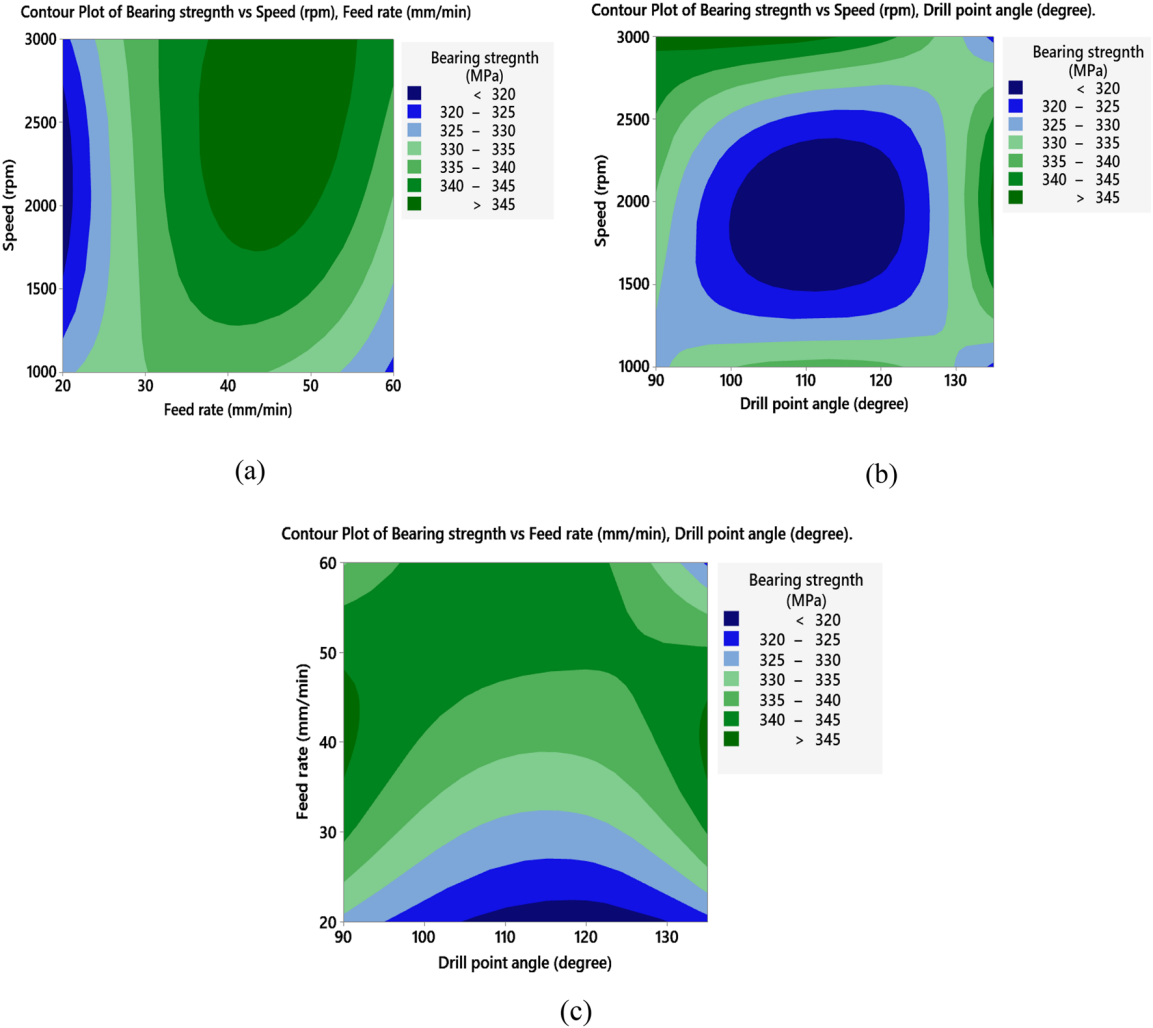
Contour plots of bearing strength for NG specimen; (**a**) speed vs feed, (**b**) speed vs drill point angle, and (**c**) feed vs drill point angle.

The S/N ratio analysis in Table 8 and Fig. 8a reveals that maximum values of bearing strength of the drilled holes in NG specimens were obtained at high spindle speed 3000 rpm, feed rate of 40 mm/min and the smallest point angle 90°. Main effect plot of means in Fig. 8b shows that, bearing strength tends to increase by raising spindle speed. Moreover, bearing strength initially increases with an increase in feed rate, but further increasing beyond 40 mm/min, leads to reduction in bearing strength. This due to increase in thrust force, which affects delamination and weakens the material's strength as described by Khashaba et al.^19^. Increasing drill point angle reduces the bearing strength this is related to small point angle produces low thrust force and hence less delamination which enhances the material strength. ANOVA analysis summarized in Table 9 shows that feed rate is the most significant factor on bearing strength with contribution of 66.60%, while spindle speed and point angle have slight contribution of 9.74% and 5.85%, respectively. The contour plot shown in Fig. 9a–c demonstrates the interaction effect of machining variables on bearing strength of NG-specimen. Effect of speed and feed rate in Fig. 9a declares that bearing strength can be maximized for spindle speed range 1500–3000 rpm and feed rate ranges between 30 and 50 mm/min. Relationship between speed and point angle, Fig. 9b, demonstrates that bearing strength is maximum at speed above 2500 rpm and point angle ranges between 90° and 120°. While Fig. 9c which shows the relationship between feed rate and point angle clears that the maximum values of bearing strength can be obtained at feed ranges between 40 and 50 mm/min and point angle below 120°.

**Table 8:** Response table for the S/N ratios (larger is better) for bearing strength.

Specimen	Level	Speed (N)	Feed rate (F)	Drill point angle (Ɵ)
NG-specimen	1	50.36	50.18	**50.54**
	2	50.43	**50.70**	50.41
	3	**50.55**	50.46	50.40
	Delta	0.19	0.52	0.14
	Rank	**2**	**1**	**3**
AG-specimen	1	49.32	**49.73**	**50.07**
	2	**49.83**	49.58	49.27
	3	49.56	49.39	49.37
	Delta	0.51	0.34	0.80
	Rank	**2**	**3**	**1**
GA-specimen	1	49.50	49.58	**49.99**
	2	**49.77**	**49.72**	49.79
	3	49.69	49.66	49.18
	Delta	0.26	0.14	0.82
	Rank	**2**	**3**	**1**

Bold values show the optimal levels of control variables.

**Table 9:** ANOVA results with contribution of control factors effect on bearing strength.

Specimen	Source	DF	Adj SS	P-value	% contribution (%)
NG-specimen	Speed (N)	2	86.10	0.646	9.74
	Feed rate (F)	2	588.67	0.211	**66.60**
	Drill point angle (Ɵ)	2	51.74	0.753	5.85
	Error	2	157.43	–	17.80
	Total	8	–	–	100.00
AG-specimen	Speed (N)	2	453.9	0.340	20.01
	Feed rate (F)	2	178.9	0.566	7.89
	Drill point angle (Ɵ)	2	1402.3	0.143	**61.81**
	Error	2	233.7	–	10.29
	Total	8	–	–	100.00
GA-specimen	Speed (N)	2	132.39	0.698	7.53
	Feed rate (F)	2	32.99	0.903	1.88
	Drill point angle (Ɵ)	2	1286.30	0.192	**73.16**
	Error	2	306.58	–	17.44
	Total	8	–	–	100.00

Bold values show the significant contribution of the parameters.

#### (B) AG specimen

**Figure 10:**
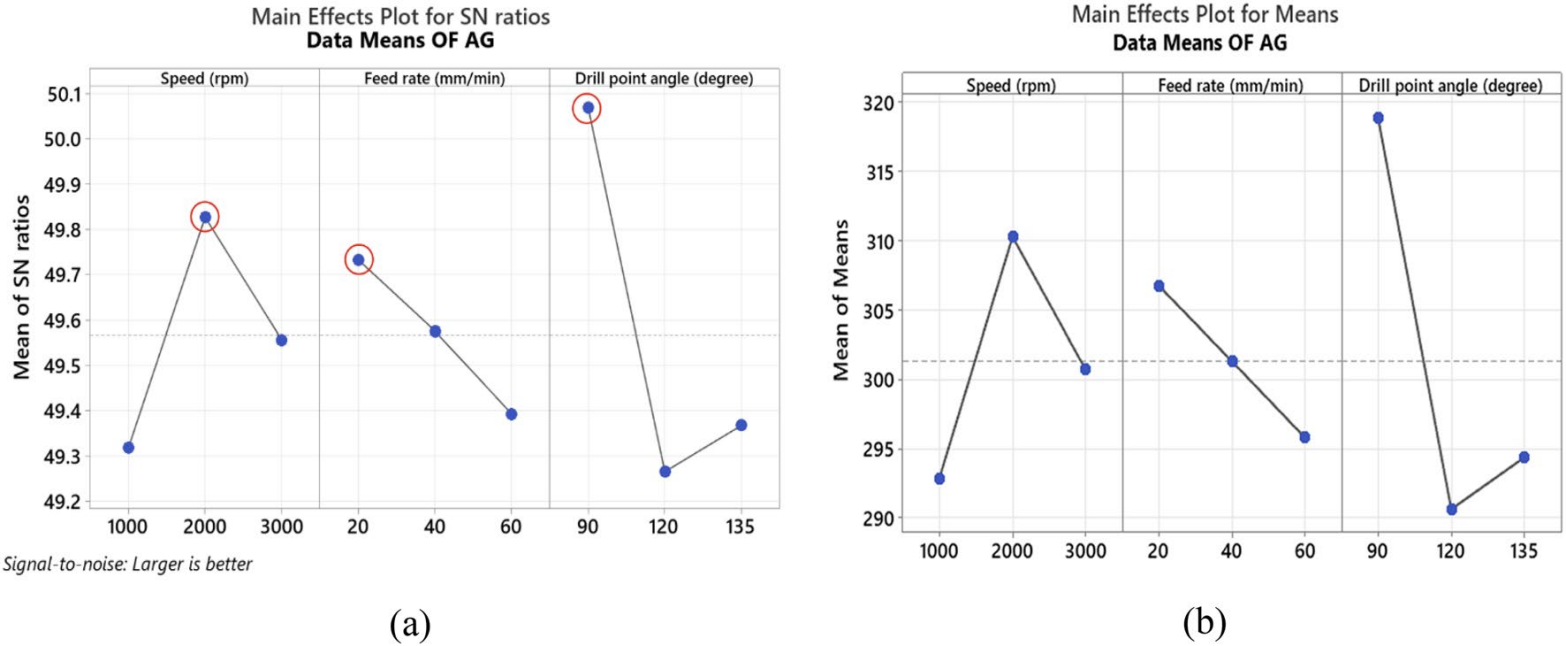
Main effects plot of bearing strength for AG specimen; (**a**) S/N ratios, and (**b**) means.

**Figure 11:**
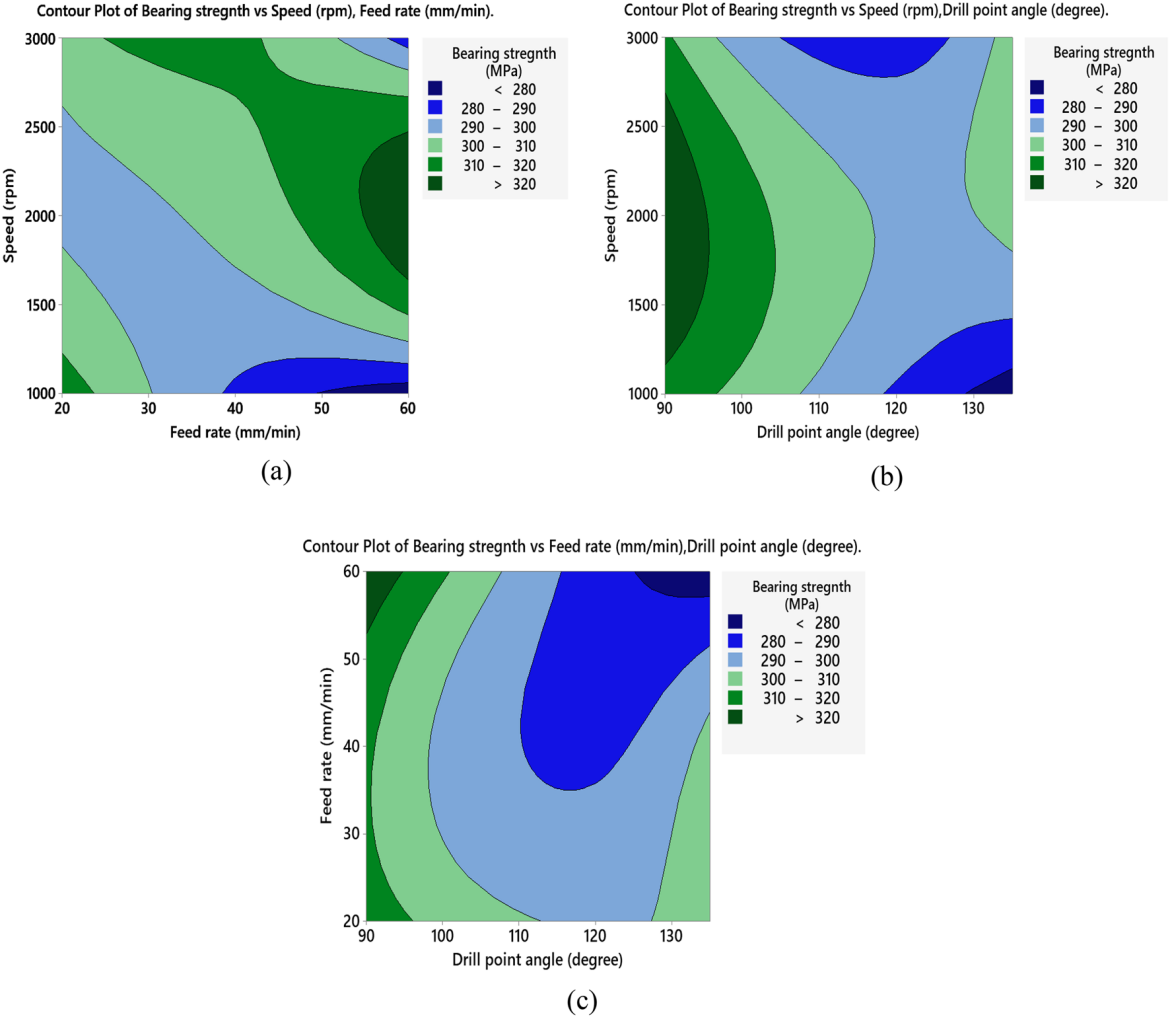
Contour plots of bearing strength for AG specimen; (**a**) speed vs feed, (**b**) speed vs drill point angle, and (**c**) feed vs drill point angle.

For AG specimen, the S/N ratio analysis listed in Table 8 and main effect of S/N response in Fig. 10a indicate that, bearing strength is maximum at spindle speed of 2000 rpm, feed rate of 20 mm/min and 90°-point angle. ANOVA analysis listed in Table 9, declares that drill point angle is of a main effect on bearing strength with contribution of 61.81% followed by spindle speed (20.01%) and insignificant effect of feed rate (7.89%). The main effect plot of means shown in Fig. 10b demonstrates that bearing strength decreases by increasing of feed rate, this occurs due to increasing thrust force and hence induced delamination^18^. Also, increasing spindle speed increases bearing strength until 2000 rpm, after which it decreases. This tendency could be attributed to the raised temperature during drilling as discussed by Khashaba et al.^19^, high generated temperature reduces thrust force and hence delamination factor, resulting in improved bearing strength than that of low-speed drilling. But further increasing of temperature above transition temperature ${T}_{g}$ of fiber leads to thermal damages and hence cause reduction in bearing strength. The small point angle of the drill (90°) improves bearing strength capacity as it has been found that drilling AG-specimen is enhanced by a low point angle, moderate spindle speed, and low feed rate. This is consistent with the findings of Sakthivel et al.^20^. The contour plot shown in Fig. 11a indicates that the bearing strength is maximum at speed ranges between 2000 and 2500 rpm and feed rate ranges between 40 and 60 mm/min. As shown in Fig. 11b, the maximum values of bearing strength are found for spindle speeds between 1500 and 2500 rpm and drill point angles less than 110°, which is in accordance with the previously disregarded influence of produced temperature. Figure 11c provides that maximum bearing values are obtained at drill point angle below 100° overall feed range.

#### (C) GA specimen

**Figure 12:**
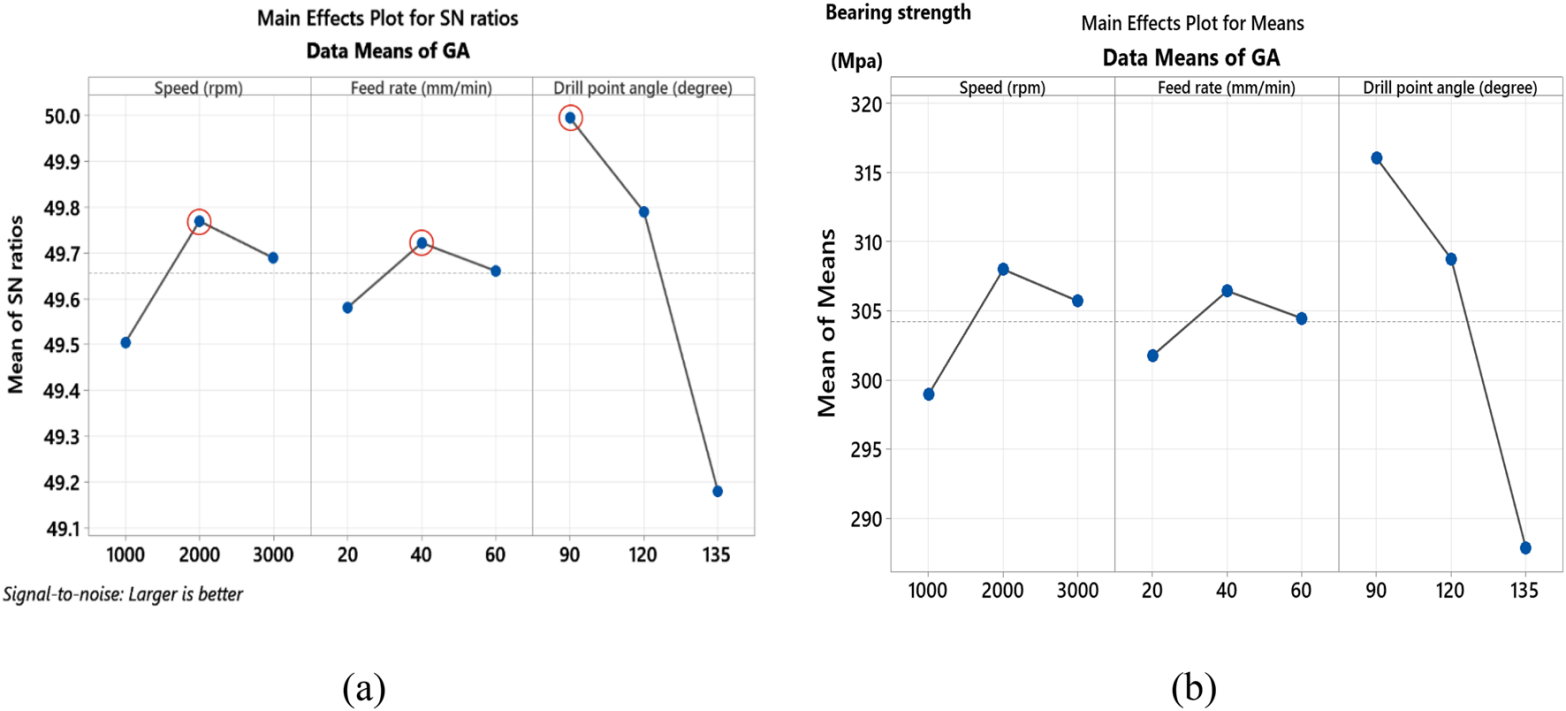
Main effects plot for GA specimen; (**a**) S/N ratios, and (**b**) means of bearing strength.

**Figure 13:**
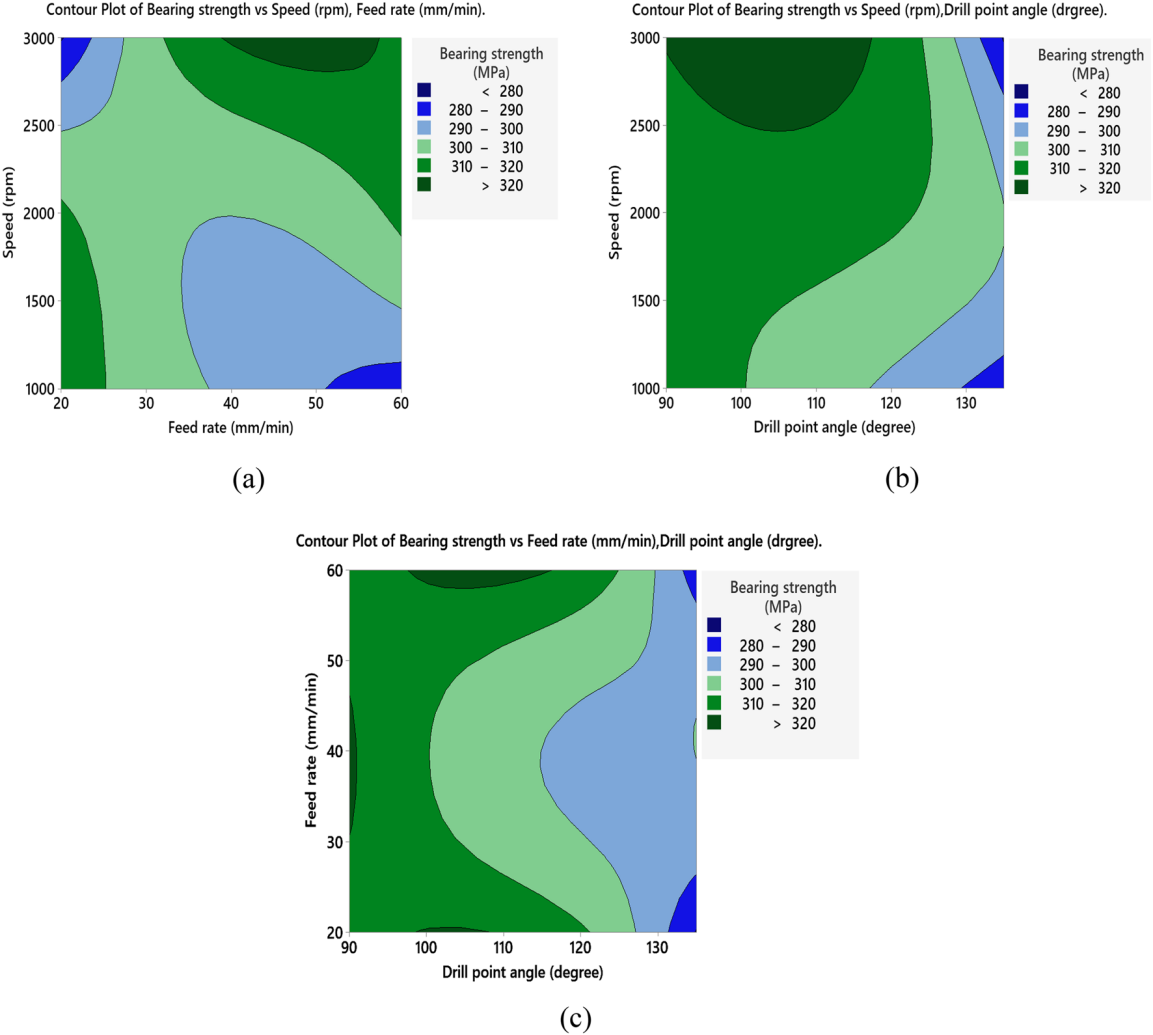
Contour plots of bearing strength for GA specimen; (**a**) speed vs feed, (**b**) speed vs drill point angle, and (**c**) feed vs drill point angle.

Table 8 clears the influence of process parameters on bearing strength. The drill point angle has the major effect on bearing strength, followed by spindle speed and feed rate has no significant influence. These results agree well with ANOVA analysis listed in Table 9. Figure 12a shows the main effects plot for S/N ratios which indicates that optimum machining parameters are spindle speed of 2000 rpm, 40 mm/min-feed rate and point angle of 90°. Main effects plot for means shown in Fig. 12b, declares that increasing of spindle speed will increase bearing strength up to 2000 rpm beyond this value bearing strength tends to decrease. Decreasing point angle increases the bearing strength, while increasing feed rate will slightly increase bearing strength but further increase will reduce the strength. This may be related to that small drill point angle, moderate speed and feed rate produce minimum delamination and hence improve bearing strength of the drilled holes in GA specimen. The contour plot in Fig. 13a demonstrates that maximum values of bearing strength are located at feed rate below 30 mm/min and spindle speed from 1000 to 2000 rpm as well as feed rate 40–50 mm/min and speed 2500–3000 rpm. Figure 13b indicates that utilizing spindle speed from 2000 to 3000 rpm and drill point angle below 110° resulting in maximum values of bearing strength. While Fig. 13c declares that maximum values of bearing are obtained at drill point angle between 90° and 100° overall feed rate range.

### Relation between bearing strength and delamination damage

**Figure 14:**
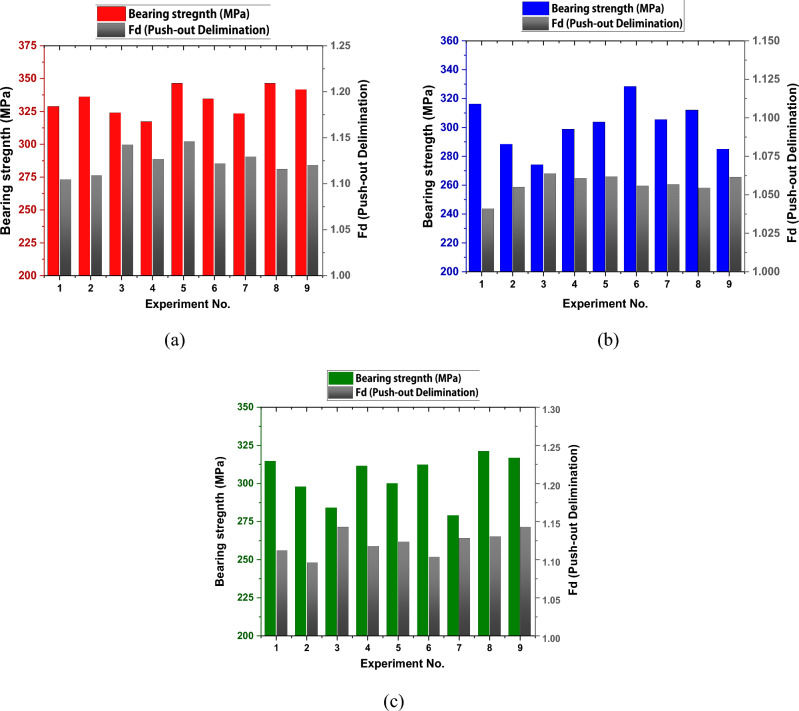
Relation between bearing strength and delamination damage for; (**a**) NG specimen, (**b**) AG specimen, and (**c**) GA specimen.

It can be seen from the relationship between bearing strength and delamination in Fig. 14 that bearing strength was increased significantly in test specimens with minimal delamination damages. In Fig. 14a maximum values of bearing strength, for NG specimen, were related to experiments 9,8,2, and 1 where delamination had small values, except experiment 5 in which bearing strength was exceptionally high. The same behavior can be seen in Fig. 14b, c for AG and GA specimens, respectively, where specimens with significant delamination damage tends to weaken the structures by concentrating too much stress at the hole edge and increasing the risk of rapid matrix cracking^38^.

### Bearing strength and failure mode investigation

**Figure 15:**
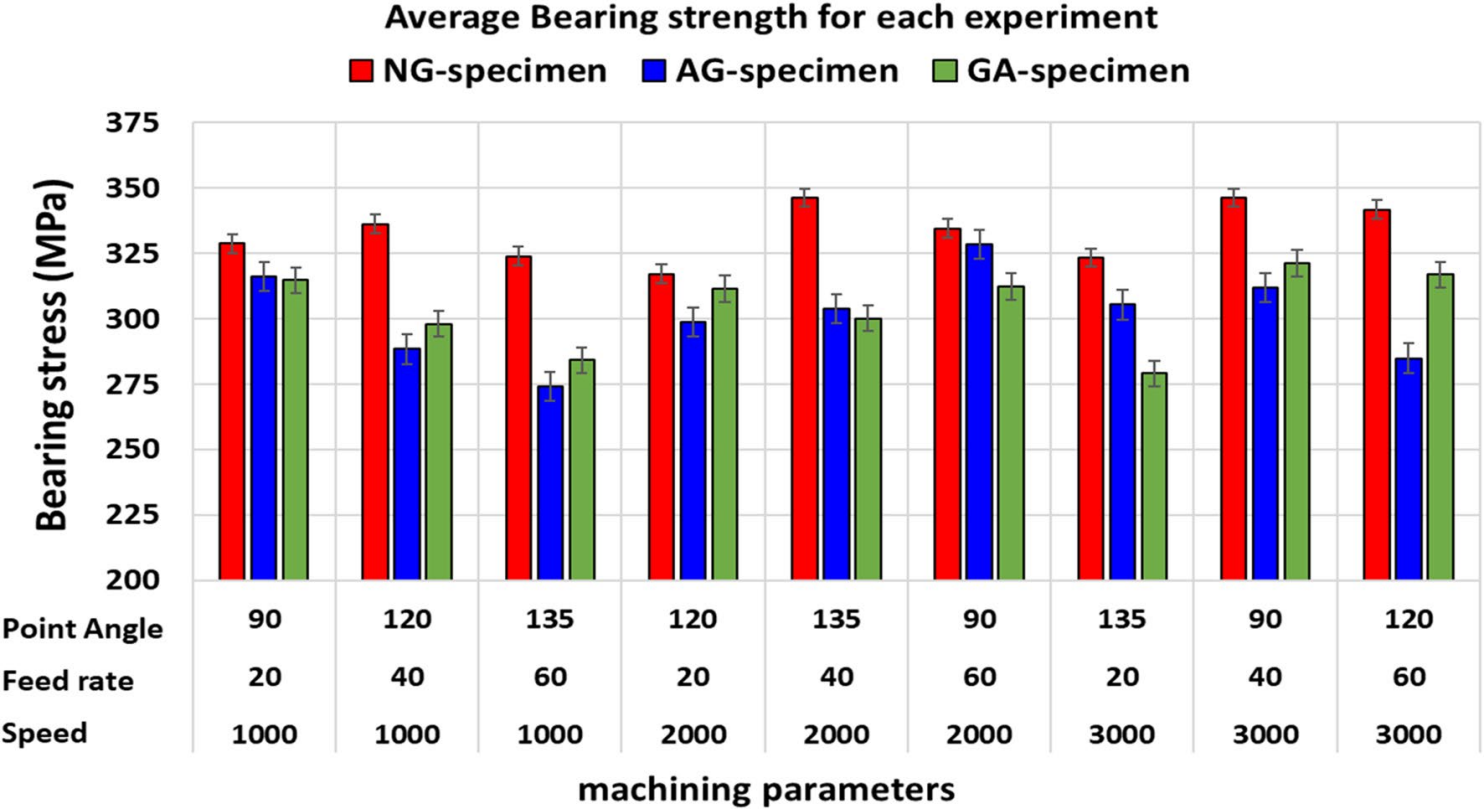
The average value of bearing strength for different specimens for each experimental setup.

From the comparison of average bearing strengths of the three specimens that are displayed in Fig. 15 it can be indicated that, NG-specimen has the highest bearing strength compared with AG and GA specimens by average percentages of 9.6% and 8.7%, respectively. This is related to the higher intrinsic tensile strength of glass fiber (NG-specimen) compared with the relatively smaller intrinsic strength of aluminum (AG and GA specimens). The average bearing strength for both AG and GA specimens is nearly identical, but AG specimen exhibits lesser delamination damage during drilling process which enhances the bearing strength of the drilled hole specimens. According to the previously indicated comparison, AG specimen results in an improvement in both peel-up and push-out delamination which enhances bearing strength. This result is evidently shown in Figs. 14 and 15 and clearly noted in photographs of Fig. 7.

**Figure 16:**
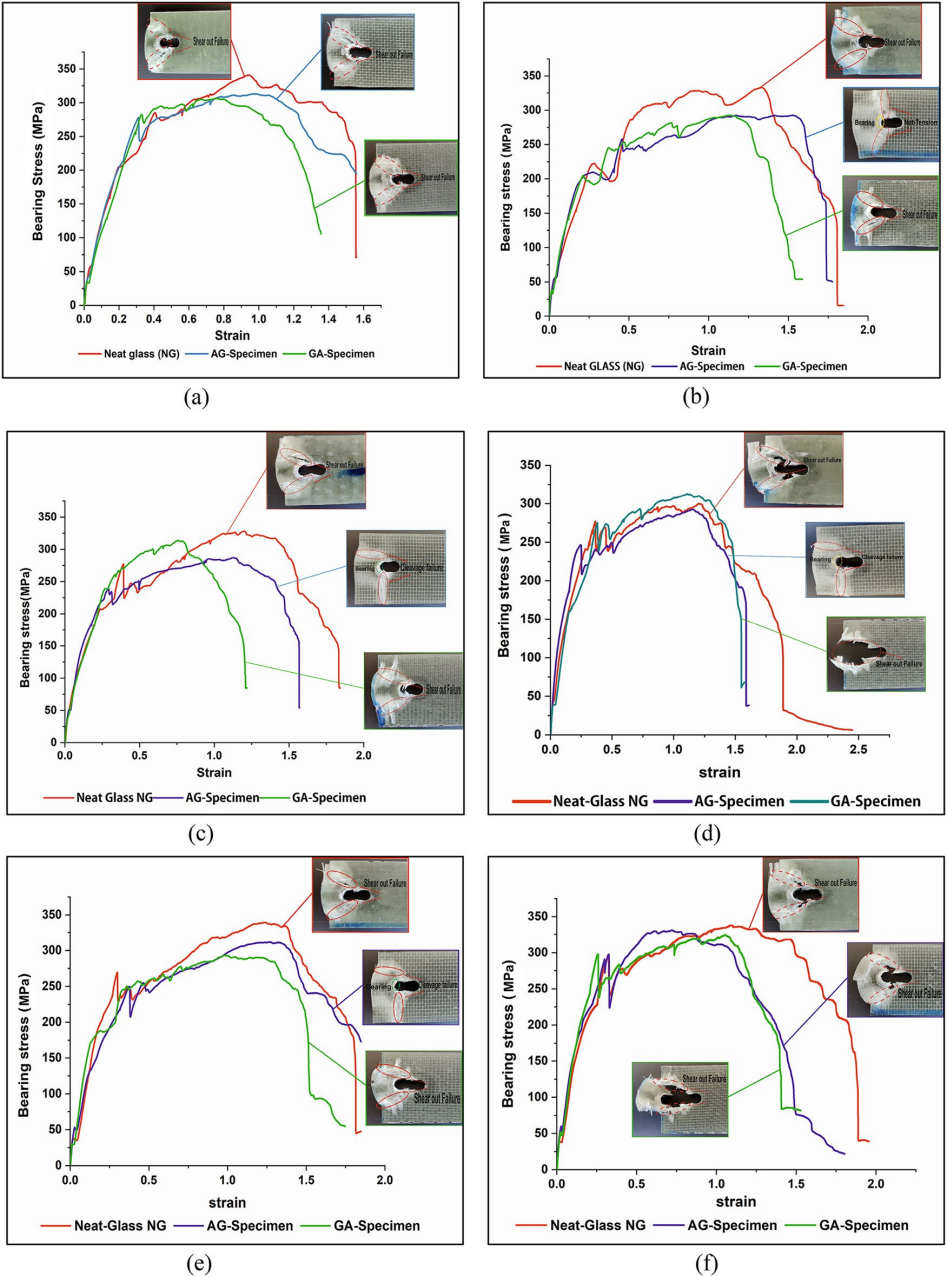

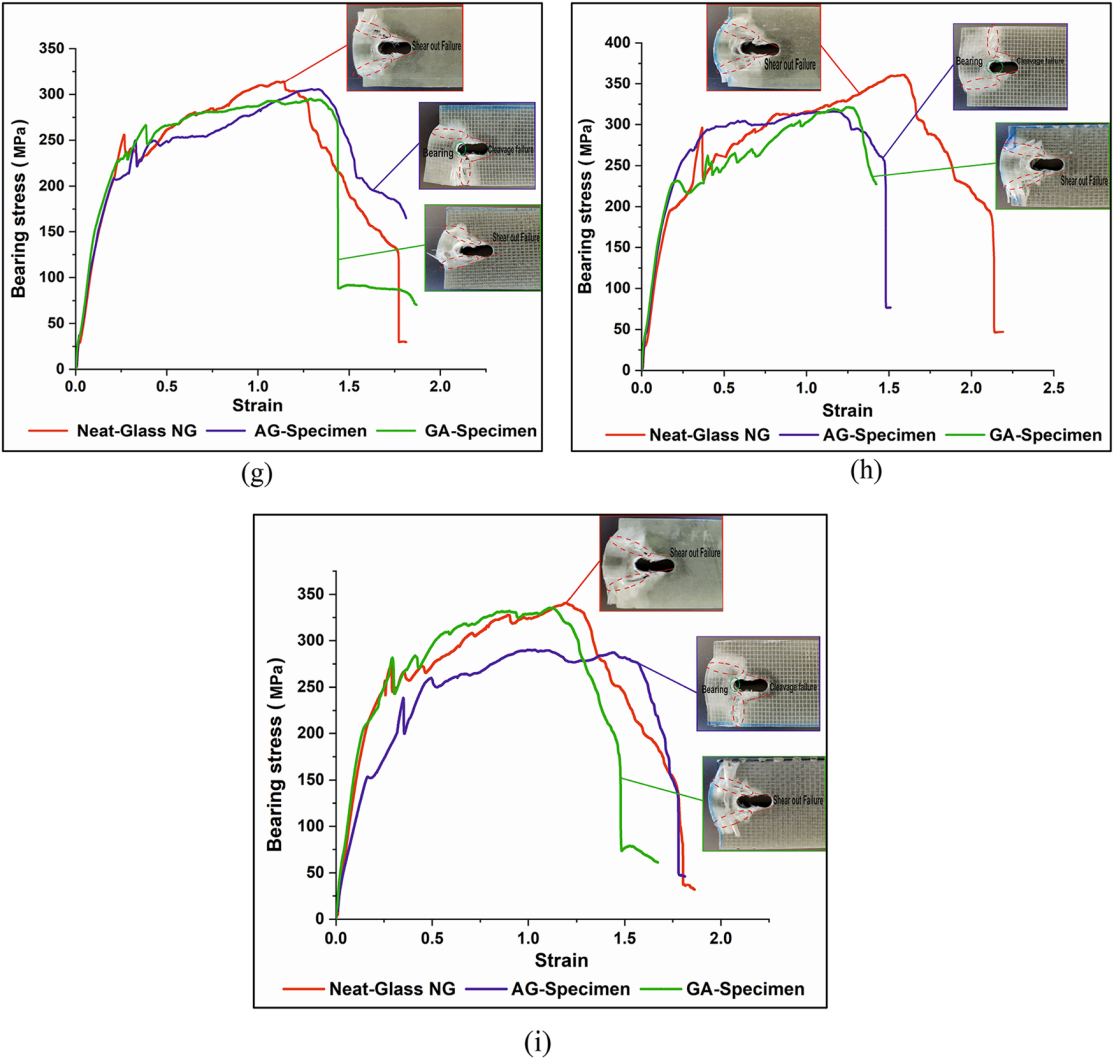
Comparison of bearing stress–strain curves of test specimens and the observed failure mode morphologies for each experiment; (**a**) exp no. 1, (**b**) exp no. 2, (**c**) exp no. 3, (**d**) exp no. 4, (**e**) exp no. 5, (**f**) exp no. 6, (**g**) exp no. 7, (**h**) exp no. 8, and (**i**) exp no. 9.

The bearing stress–strain curves and failure modes shown in Fig. 16 demonstrates that, shear-out failure is the most failure type due to a short distance from the edge e/d = 3. In case of NG and GA specimens, shear-out failure mode exists in all bearing tests. Shear-out failure is generally caused by matrix and fiber shear and compression failures^39^, in which high shear stress formed at the shear-out plan could not be released by the free edge distance^40^. In the case of AG specimen, three various failure mechanisms including shear-out, cleavage, and net-tension failure can be experimentally observed. The cleavage mix mode is the most existed failure in AG specimen. Cleavage failure is produced by the load concentration at the hole tip and fastener's bearing pressure. In AG specimen, shear-out failure mode is remarked by the high bearing strength as for experiments 1 and 6.

### Confirmation tests of Taguchi optimization

After the levels of the drilling variable combinations that yielded the best performance were attained. A confirmation test was needed to validate the optimized condition when using the Taguchi technique^41^. The predicted responses can be determined using Eq. (6)

6 $${y}_{opt}={y}_{m}+\left({N}_{opt}-{y}_{m}\right)+\left({F}_{opt}-{y}_{m}\right)+ \left({\theta }_{opt}-{y}_{m}\right)$$

where, ${y}_{opt}$ is the estimated optimum value of delamination factor and bearing strength, respectively. (${N}_{opt}$, ${F}_{opt}$ and ${\theta }_{opt}$) represent the mean value for each variable at the optimum condition for each response are represented in Table 10. ${y}_{m}$ States the total mean value of each response which is obtained from experimental study listed in Table 5. Table 11 shows the results of confirmation experiments. The experimental and estimated values are quite similar. Error levels must be under 20% for analysis to be considered reliable^42^. It is noted that several optimum parameter configurations do not match with any experimental trial in Table 5. Using the same procedure as described in section "Drilling process", four test specimens were drilled by the optimal parameters attained by Taguchi. According to section "Assessment of the responses", the delamination factor assessment and bearing strength test was performed five times, and the average value was determined.

**Table 10:** Mean response values for each variable at the optimum condition for each response.

Specimen	Response	Total mean value, ${y}_{m}$	Process variables		
			Speed (N)	Feed rate (F)	Drill angle (Ɵ)
NG-specimen	Peel-up delamination	1.09413	1.0854	1.086	1.088
	Push-out delamination	1.12368	1.1182	1.1999	1.11379
	Bearing strength	333.081	337.1	342.9	336.5
AG-specimen	Peel-up delamination	1.0564	1.0563	1.0538	1.0502
	Push-out delamination	1.0582	1.05306	1.0526	1.0501
	Bearing strength	301.29	310.26	306.7	318.816
GA-specimen	Peel-up delamination	1.1114	1.1033	1.09105	1.1088
	Push-out delamination	1.1216	1.1146	1.1164	1.1148
	Bearing strength	304.22	308	306.44	316.054

**Table 11:** Results of confirmation tests by Taguchi method.

Specimen	Response	Process variables		
		Exp	Pred	Error (%)
NG-specimen	Peel-up delamination	**1.0789**	1.07114	0.72
	Push-out delamination	1.10419	1.18453	7.27
	Bearing strength	346.23	350.338	1.18
AG-specimen	Peel-up delamination	1.03877	1.0475	0.84
	Push-out delamination	1.04081	1.03936	0.14
	Bearing strength	**312.15**	333.196	6.74
GA-specimen	Peel-up delamination	1.08016	1.08035	0.017
	Push-out delamination	**1.1164**	1.1026	1.23
	Bearing strength	**307.89**	322.054	4.60

Bold values show the estimated experimental values of the prepared specimen.

### Regression analysis

Regression analysis was used to model the correlation between the drilling parameters and the response factors^43^. The multiple model which was used to forecast how the response factor would be affected by the machining settings is given by Eq. (7).

7 $$Y= {B}_{0}+{B}_{1}{X}_{1}+{B}_{2}{X}_{2}+{B}_{3}{X}_{3}+\dots +{B}_{n}{X}_{n}$$

where *Y* is the response, *B*’s are the actual weighting factors and *X’s* are the independent variables.

The final equation which describes the interaction of the four variables in the study is given by Eq. (8).

8 $${\sigma }_{bearing}= a+{b}_{1}N+{b}_{2}F+{b}_{3} \uptheta$$

where ${\sigma }_{bearing}$ is the response bearing strength, $N$ is spindle speed, $F$ is feed rate and $\uptheta$ is the drill point angle. By substituting values into the regression equation and resolving the equations, the values of *a*, *b*_*1*_, *b*_*2*_, and *b*_*3*_ can be determined^44^. Equations (9) and (10) are used to determine the model accuracy according to the mean absolute percentage error (MAPE) and prediction accuracy (PA) as given:

9 $$\text{The } \,(\text{MAPE}) \,\text{ of \, the \, model } \,= \,\frac{1}{n}{\sum }_{1}^{n}\left|\frac{\text{Exp.value } - \text{ Pred.value}}{\text{Exp.value}} \times 100\right|$$

10 $$\text{Prediction \,accuracy } \,(\text{PA}) = 100-\text{MAPE}$$

where *n* is the number of experiments, $\text{Exp}.\text{value}$ is the experimental value, and $\text{Pred}.\text{value}$ is the predicted value.

**Figure 17:**
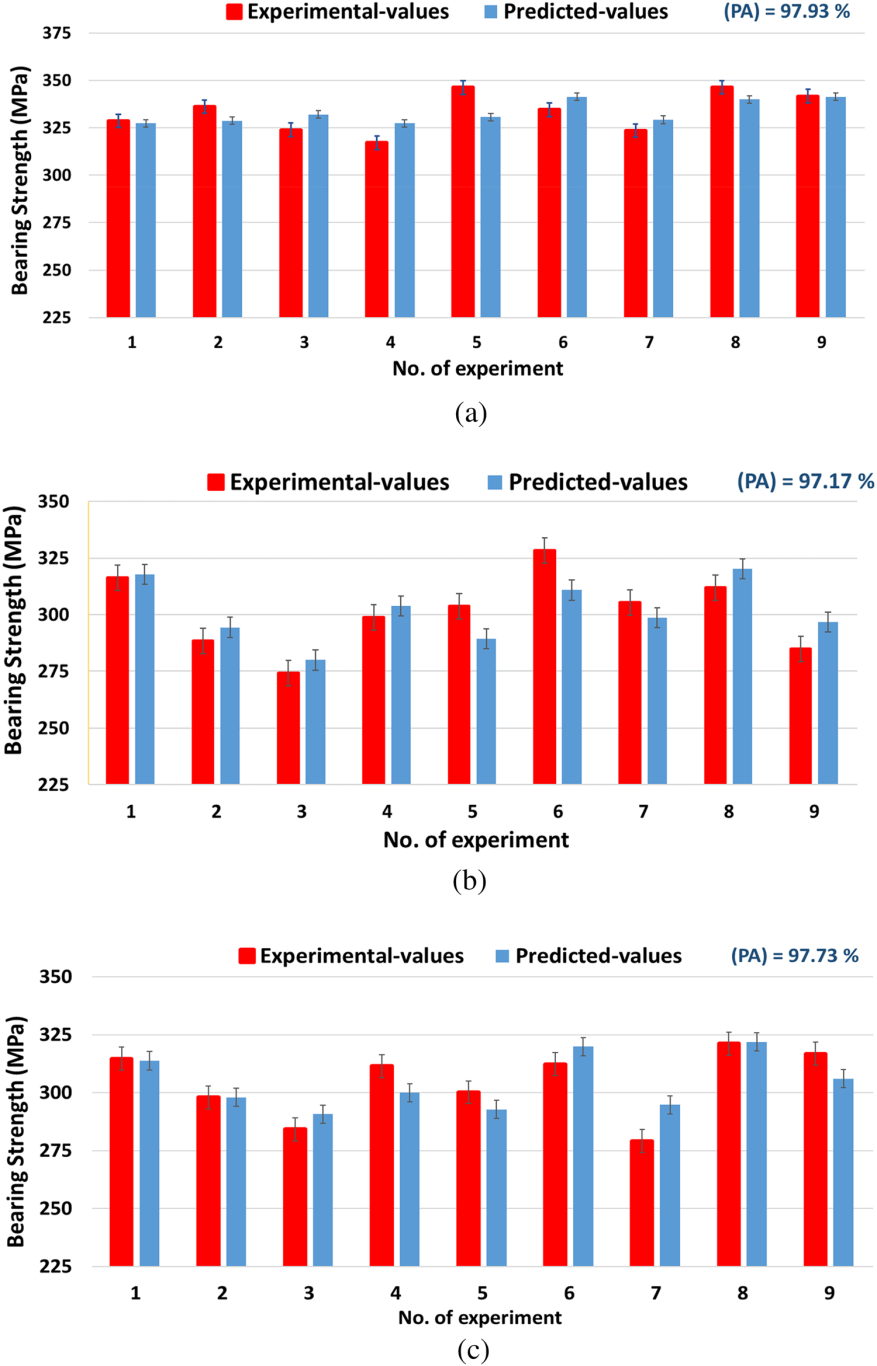
Validation of experimental and predicted values of Bearing strength for; (**a**) NG-specimen, (**b**) AG-specimen, (**c**) GA-specimen.

The regression equations of bearing response are listed in Table 12. The calculated data and mean error are listed in Table 13 for NG, AG, and GA specimens, respectively. From the results of Table 13 it was found that regression equations can provide remarkable agreement between experimental data and predicted results. Validation of experimental and predicted results for bearing strength response of the specimens is displayed in Fig. 17.

**Table 12:** Regression equations for bearing strength.

Response	Regression equation
${\sigma }_{NG}$	$329.5+3.76{\times 10}^{-3} N+0.257 F-0.124 \uptheta$
${\sigma }_{AG}$	$373.2841429+3.911{\times 10}^{-3} N-0.27305 F-0.5990254 \uptheta$
${\sigma }_{GA}$	$360.56119+0.003371 N+0.067249 F-0.571952{ \uptheta}$

*N* spindle speed, $F$ feed rate, *Ɵ* point angle.

**Table 13:** Experimental, predicted, and mean error values (%) of bearing strength.

Validation of data for bearing strength									
No	NG specimen			AG specimen			GA specimen		
	Experimental value	Predicted value	MAPE %	Experimental value	predicted value	MAPE %	Experimental value	Predicted value	MAPE %
1	328.69	327.27	0.43	316.15	317.82	0.53	314.68	313.80	0.279
2	336.14	328.70	2.21	288.4	294.39	2.08	297.98	297.99	0.003
3	323.89	331.98	2.50	274.15	279.94	2.11	284.22	290.75	2.299
4	317.07	327.31	3.23	298.7	303.76	1.69	311.49	300.02	3.684
5	346.19	330.60	4.50	303.7	289.32	4.73	300.18	292.78	2.465
6	334.46	341.33	2.05	328.4	310.81	5.36	312.32	319.86	2.415
7	323.39	329.21	1.80	305.36	298.69	2.19	279.13	294.81	5.617
8	346.23	339.94	1.82	311.9	320.18	2.65	321.15	321.89	0.231
9	341.67	341.37	0.09	284.9	296.75	4.16	316.83	306.08	3.394
The (MAPE) of the model			2.07	The (MAPE) of the model		2.83	The (MAPE) of the model		2.265
Prediction accuracy (PA)			97.93%	Prediction accuracy (PA)		97.17%	Prediction accuracy (PA)		97.73%

## Conclusions

CNC drilling of neat GFRE and hybrid GFRE/Al-wire mesh specimens was investigated in this work to determine the influence of drilling parameters and induced delamination on the bearing strength of the tested specimens. The optimization and influence of the drilling parameters was performed by utilizing Taguchi and ANOVA analyses. The outcomes from this study are outlined below:

- The maximum values of bearing strength of the drilled holes were obtained at point angle of 90°, feed rate (*F*) = 40 mm/min for NG and GA specimens and *F* = 20 mm/min for AG specimen, and spindle speed (*N*) = 2000 rpm for both AG and GA specimens and *N* = 3000 rpm for NG specimen.
- In the case of NG specimen feed rate has the major effect on the bearing strength with contribution of 66.60%, while for both AG and GA specimens the drill point angle is the most influential parameter of 61.81% and 73.16% contribution, respectively.
- The bearing strength of the specimen was significantly affected by the induced delamination of the drilled hole. The bearing strength was decreased with increasing the delamination factor.
- The lowest delamination existed in AG specimen increases its bearing strength, which was nearly equivalent to GA's average bearing strength.
- For NG and GA specimens, the shear-out failure mode exists in all specimens. While for AG specimen, three failure mechanisms can be experimentally observed, including shear-out, cleavage, and net-tension. The cleavage mix mode is the most observed failure mode.
- Adding Al mesh to the outer layers (AG specimen) improves drilling quality and enhances the load bearing capacity, revealing that it can be employed in advanced applications like aerospace and automobiles sectors.
- It was found that regression equations can provide remarkable agreement between experimental data and predicted results.

## Data Availability

The datasets used and analyzed during the current study are available from the corresponding author on reasonable request.
